# Efficacy of Radio Electric Asymmetric Conveyer (REAC) Biomodulation Treatments in Managing Chronic Pain, Edema, and Metabolic Syndrome: A Case Report

**DOI:** 10.7759/cureus.72031

**Published:** 2024-10-21

**Authors:** Ludmilla Higino Rocha, Vania Fontani, Salvatore Rinaldi

**Affiliations:** 1 Anesthesiology, International Scientific Society of Neuro Psycho Physical Optimization With Radio Electric Asymmetric Conveyer (REAC) Technology, São Paulo, BRA; 2 Research, Rinaldi Fontani Foundation, Florence, ITA; 3 Regenerative Medicine, Rinaldi Fontani Institute, Florence, ITA

**Keywords:** chronic pain management, chronic systemic inflammation, circulatory optimization, metabolic optimization, metabolic syndrome, neurovegetative dysfunction, reac biomodulation, tissue reparative therapy

## Abstract

This case report presents a 67-year-old male with a history of metabolic syndrome, uncontrolled diabetes mellitus, hypertension, and prostate cancer, who underwent radio electric asymmetric conveyer (REAC) biomodulation treatments, specifically anti-cellulite treatment (ACT), circulatory optimization (CO), and metabolic optimization (MO). The patient initially presented with severe pain and edema in the left lower limb, requiring morphine. Over three months, he received a combination of ACT, CO, and MO protocols. Remarkable improvements were observed in pain intensity, edema reduction, balance, and overall quality of life, with a reduced reliance on morphine. This report highlights the potential of REAC biomodulation therapies in managing complex conditions associated with metabolic syndrome.

## Introduction

Metabolic syndrome is a multifaceted condition characterized by a combination of risk factors [1], including central obesity, insulin resistance, dyslipidemia, hypertension, and inflammatory disorders [2], which collectively increase the risk for cardiovascular disease, type 2 diabetes mellitus, and other health complications [3]. Patients with metabolic syndrome often present with chronic pain [4], edema, reduced mobility, and diminished quality of life [5], making effective management challenging, especially in those with comorbidities such as prostate cancer or a history of deep vein thrombosis.

Traditional treatment approaches for metabolic syndrome primarily focus on lifestyle modifications, pharmacological interventions, and managing individual symptoms [6]. However, these strategies may not adequately address the underlying systemic dysregulation [7] or provide sufficient relief from chronic pain and edema.

Radio electric asymmetric conveyer (REAC) treatments aimed at promoting reparative effects are collectively referred to as tissue optimization reparative (TO-RPR), followed by the specific indication for each protocol. Depending on the dysfunction or lesion being treated, these protocols are applied in succession, as demonstrated in this case report. In this case, we utilized the REAC anti-cellulite treatment (TO-RPR ACT) protocol, which promotes tissue health and addresses chronic inflammation, a key factor in persistent pain and impaired skin function in metabolic syndrome [8]. By targeting this inflammation, the ACT protocol not only improves skin texture but also reduces pain. The circulatory optimization (TO-RPR CO) protocol enhances blood flow, reduces edema, and supports lymphatic drainage, addressing the circulatory challenges often associated with metabolic syndrome [9]. Additionally, the metabolic optimization (TO-RPR MO) protocol restores metabolic balance, improves energy efficiency, and supports weight reduction, which are crucial components in managing metabolic syndrome. Together, these REAC treatments provide an integrated restorative approach, enhancing patient outcomes through targeted cellular and tissue repair [10-13]. The treatment involved positioning the interconnected asymmetric conveyor probes, which were attached to the BENE 110 device, on the patient's right and left femoral quadriceps.

## Case presentation

The patient is a 67-year-old male with a complex medical history that includes metabolic syndrome, poorly controlled type 2 diabetes mellitus, hypertension, and a diagnosis of prostate cancer in 2018. His diabetes and hypertension have been difficult to manage, leading to several complications. One of the most prominent complications has been chronic pain, particularly in his left lower limb, where he also suffers from significant recurrent edema. The swelling and discomfort in this area have been severe enough to necessitate the intermittent use of morphine during flare-ups to manage the pain.

The patient has also reported occasional muscle cramps, though these have not been persistent. Despite his complex medical condition, he describes his sleep patterns as good and initially reported a stable mood, denying anxiety or depression. However, during the first evaluation, his actual attitude appeared discordant with this self-assessment. His behavior and emotional state suggested a more subdued or possibly distressed mood that did not align with his verbal statements about his psychological well-being. He is a former smoker and alcohol consumer, having quit both habits four years ago. He is not currently taking any medications beyond those prescribed for his chronic conditions and denies any known medication, food, or environmental allergies.

Clinical findings

The patient presented with generalized hypotonia, characterized by reduced muscle tone and strength. There was significant abdominal obesity, with a weight of 101 kg and a height of 1.68 m, resulting in a BMI of 35.8 kg/m², indicating class II obesity. The abdominal circumference measured 124 cm, further confirming central adiposity. The patient reported severe pain in the left lower limb, scoring 10/10 on the visual analog pain scale, indicating the maximum level of pain intensity.

Physical examination revealed marked edema in the lower limbs, particularly on the left side, accompanied by erythema. The patient demonstrated significantly reduced mobility and was unable to perform daily activities without assistance. Additionally, gait assessment showed a pronounced limp, and the patient frequently required support for ambulation due to pain and weakness. These findings suggest the involvement of both inflammatory and mechanical factors contributing to the patient's impaired mobility and quality of life.

Details on REAC TO-RPR ACT, CO, and MO protocols

All therapeutic protocols administered using medical devices based on REAC technology, such as the BENE 110 (ASMED, Scandicci, Italy), operate with specific pre-set parameters that ensure the repeatability and effectiveness of each treatment. These parameters cannot be adjusted by the operator, which guarantees consistent therapeutic outcomes.

The REAC TO-RPR ACT, CO, and MO protocols follow structured cycles, with each cycle consisting of 18 sessions, and each session lasting approximately 15 minutes. Sessions can be spaced out by a minimum of one hour and up to one week apart, with no more than four sessions administered in a single day. Importantly, these protocols must be performed in succession: the ACT protocol is completed first, followed by the CO protocol, and finally the MO protocol. This sequential approach maximizes the therapeutic benefits.

The REAC TO-RPR ACT, CO, and MO protocols work synergistically to target systemic issues such as inflammation, circulation, and metabolic dysfunction, resulting in an integrated and restorative treatment plan tailored to enhance overall patient outcomes.

Therapeutic interventions

The ACT was initiated on July 24, 2024, and the patient completed 18 sessions within one week. The treatment yielded notable improvements, including enhanced skin appearance, a marked reduction in edema, and a weight loss of 2.2 kg. Additionally, the patient reported decreased pain intensity, allowing him to discontinue the use of morphine for pain management.

On August 1, 2024, the CO protocol was introduced, consisting of 18 sessions over a two-week period. Following this treatment, the patient experienced a significant improvement in mood, further reductions in pain intensity, and increased mobility. His physical therapist also observed substantial decreases in lower limb edema and erythema, highlighting the positive impact of the protocol on his overall circulatory health.

The MO treatment commenced on August 13, 2024, and was administered over three weeks, with a total of 18 sessions. Throughout this period, the patient exhibited continued pain reduction, improved balance, and an enhanced sense of overall well-being. Additional benefits included better appetite control, a decrease in food cravings, and improved sleep quality, further contributing to his overall recovery and metabolic health.

Outcomes and follow-up

**Figure 1:**
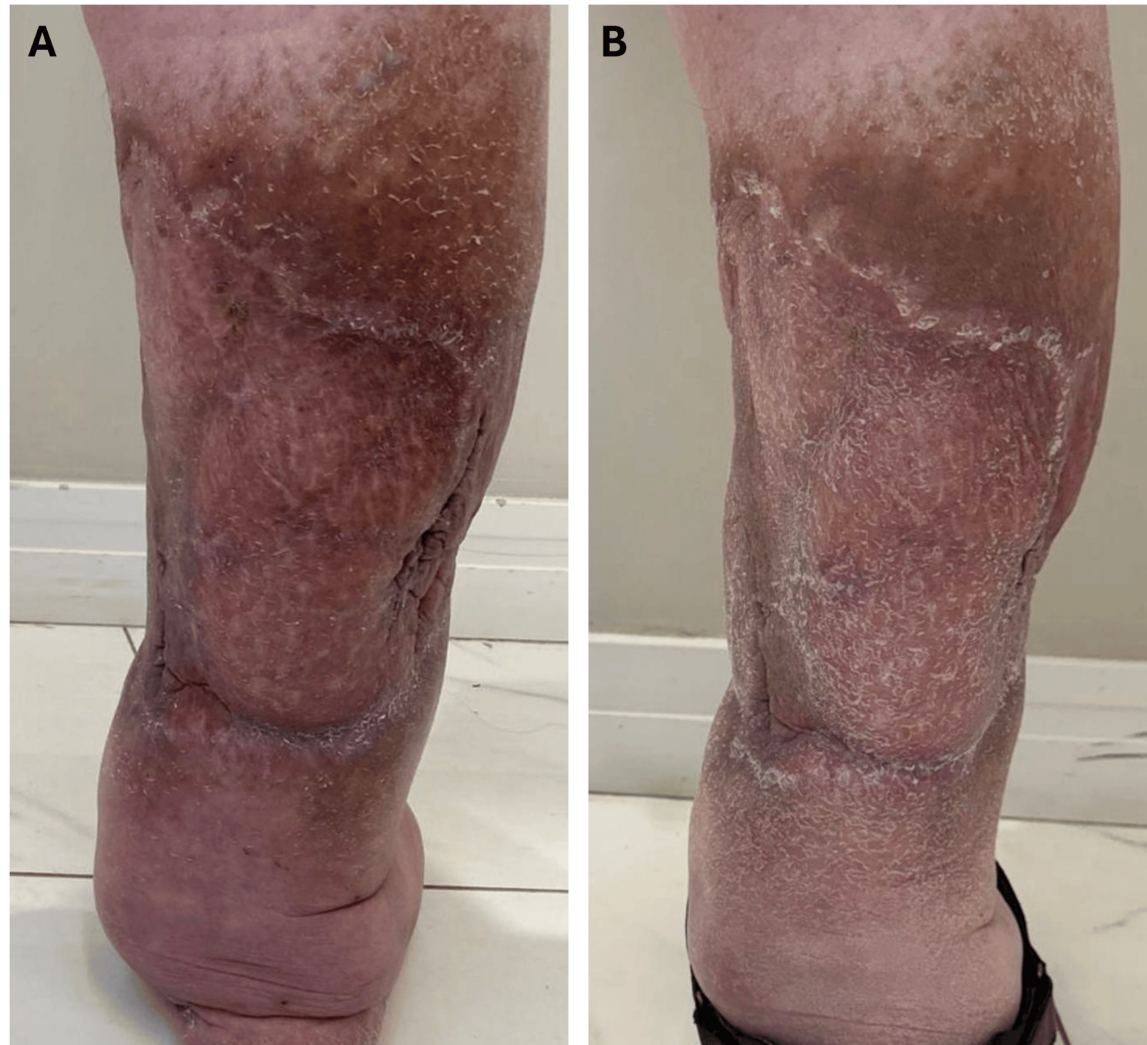
Comparative images of the patient's left lower posterior leg before and after REAC TO-RPR ACT, CO, and MO treatments. Figure 1A shows the posterior leg prior to treatment, displaying significant edema, thickened skin, and discoloration due to chronic inflammation and poor circulation. Figure 1B depicts the posterior leg after the therapy cycles, with a visible reduction in edema, improved skin texture, and normalized coloration. The duration between the 'before' and 'after' photos is approximately three months. REAC: Radio Electric Asymmetric Conveyer; TO-RPR: Tissue optimization reparative; ACT: Anti-cellulite treatment; CO: Circulatory optimization; MO: Metabolic optimization.

**Figure 2:**
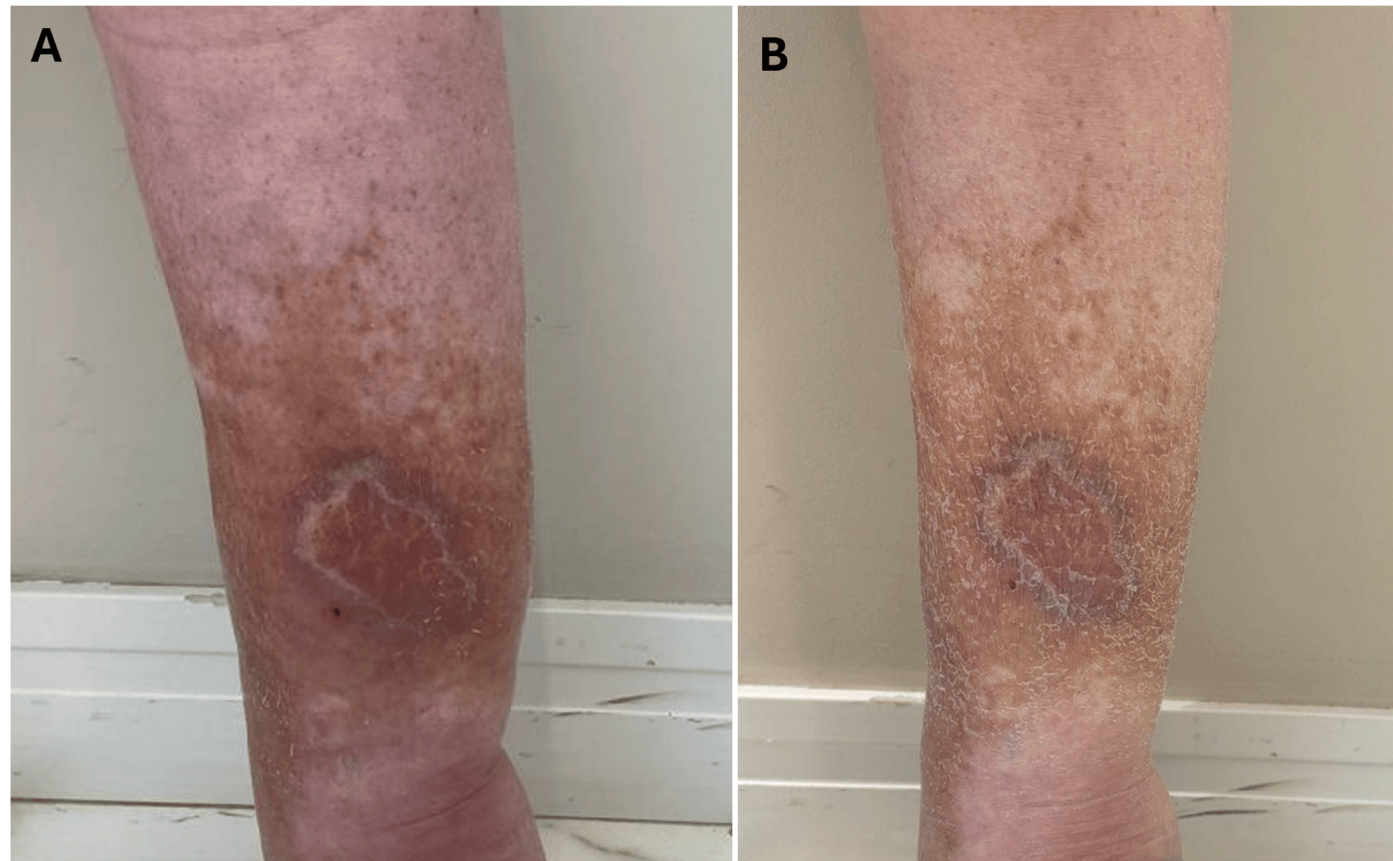
Comparative images of the patient's lower anterior left leg before and after REAC TO-RPR therapy. Figure 2A: Comparative images of the patient's left lower anterior leg before and after REAC TO-RPR therapy. Figure 2A illustrates the anterior leg before treatment, showing severe inflammation, discoloration, and a chronic wound. Figure 2B demonstrates the same anterior leg after therapy, with noticeable reductions in inflammation, improved skin quality, and significant wound healing. The duration between the 'before' and 'after' photos is approximately three months. REAC: Radio Electric Asymmetric Conveyer; TO-RPR: Tissue optimization reparative.

The combined REAC treatments resulted in substantial improvements in the patient’s condition. There was a marked reduction in pain intensity, which decreased from an initial 10/10 to 5/10 on the pain scale, indicating a significant enhancement in pain management. Additionally, a considerable reduction in lower limb edema was observed, accompanied by an improvement in skin appearance, with decreased erythema and improved elasticity (Figures 1-2).

The patient experienced notable weight loss, with a final recorded weight of 90.5 kg, amounting to a total loss of 5 kg. This weight reduction contributed to a noticeable decrease in abdominal circumference, indicating an improvement in central obesity. Furthermore, the patient reported increased energy levels and mood stability, which translated into a better overall quality of life and improved mobility. The ability to engage in daily activities without requiring support improved significantly.

Throughout the treatment period, no adverse effects were observed, and the patient successfully maintained abstinence from morphine use, which he had previously relied on for pain management. The absence of medication dependence further underscores the efficacy and safety of the REAC treatments in addressing the patient's chronic pain, edema, and metabolic challenges. The patient continues to be monitored regularly to ensure the sustained benefits of the treatment.

## Discussion

This case demonstrates the potential effectiveness of REAC biomodulation treatments, specifically the ACT, CO, and MO protocols, in managing complex and multifactorial symptoms associated with metabolic syndrome. The patient's significant improvement in pain intensity, edema reduction, and overall quality of life suggests that REAC treatments could serve as a valuable adjunct in managing conditions often resistant to conventional therapies.

The ACT protocol, which primarily targets chronic inflammation, played a crucial role in reducing pain and improving the skin's appearance. Chronic inflammation is a well-recognized component of metabolic syndrome and is often linked to the development of insulin resistance [14], obesity, and cardiovascular complications [15]. By addressing inflammation, ACT not only enhanced the patient's comfort but also contributed to the reduction in peripheral edema and pain, which are common complications in patients with metabolic disorders.

The CO protocol demonstrated further benefits in enhancing blood flow, reducing edema, and improving lymphatic drainage. Given that impaired circulation is a hallmark of metabolic syndrome, especially in patients with a history of deep vein thrombosis or other vascular complications [16], the CO protocol's impact is particularly noteworthy. The patient’s enhanced mobility and reduced reliance on pain medication during this phase underscore the importance of improving circulatory health in managing the systemic effects of metabolic syndrome.

The MO protocol was pivotal in addressing the metabolic dysregulation inherent in this patient’s condition. By improving metabolic efficiency and promoting energy balance, the MO protocol facilitated weight loss, reduced appetite, and stabilized blood glucose levels, which are critical factors in managing metabolic syndrome. The patient's ability to reduce his morphine usage and report sustained improvements in mood, sleep quality, and overall energy levels highlights the potential of the MO protocol to offer long-term benefits in metabolic regulation [17].

This case reinforces the concept that metabolic syndrome, with its diverse and interlinked pathophysiological mechanisms, may benefit from an integrative therapeutic approach targeting multiple pathways simultaneously. REAC treatments, through their capacity to influence inflammation, circulation, and metabolic processes, present a promising adjunctive therapy that goes beyond symptom management to address underlying dysfunctions. This integrative approach may be particularly advantageous in patients with complex medical histories, such as those with comorbidities like diabetes, cardiovascular issues, or a history of cancer, where standard treatments may be insufficient or limited in scope.

While this case report presents encouraging outcomes, further research with larger cohorts and controlled trials is necessary to establish the efficacy, optimal protocols, and long-term benefits of REAC biomodulation treatments. Investigating the molecular mechanisms underlying REAC's effects on cellular and tissue function could also provide insights into how these treatments can be tailored to individual patients' needs, particularly in cases with severe or treatment-resistant metabolic conditions.

In conclusion, this case supports the use of REAC biomodulation treatments as a promising therapeutic adjunct for managing metabolic syndrome and its associated complications. The notable improvements observed in this patient highlight the potential for these protocols to address multifactorial symptoms effectively, suggesting that REAC technology could be an innovative addition to the management strategies for metabolic syndrome.

## Conclusions

In conclusion, this case supports the use of REAC biomodulation treatments as a promising therapeutic adjunct for managing metabolic syndrome and its associated complications. The notable improvements observed in this patient highlight the potential for these protocols to effectively address multifactorial symptoms, suggesting that REAC technology could be an innovative addition to management strategies for metabolic syndrome. Further studies are warranted to better understand its role and potential benefits in broader clinical practice.
